# Climate change and livestock herders wellbeing in Pakistan: Does nexus of risk perception, adaptation and their drivers matter?

**DOI:** 10.1016/j.heliyon.2023.e16983

**Published:** 2023-06-03

**Authors:** Muhammad Usman, Asghar Ali, Joanna Rosak-Szyrocka, Ladislav Pilař, Sajjad Ahmad Baig, Rimsha Akram, Abdulazeez Hudu Wudil

**Affiliations:** a Institute of Agricultural and Resource Economics, University of Agriculture, Faisalabad, Pakistan; b Faculty of Management, Czestochowa University of Technology, 42-200, Czestochowa, Poland; c Department of Management, Faculty of Economics and Management, Czech University of Life Sciences Prague, Prague, Czech Republic; d Faisalabad Business School, National Textile University, Faisalabad, Pakistan; e Department of Botany, University of Agriculture, Faisalabad, Pakistan; f Department of Agricultural Economics and Extension, Federal University, Dutse, Jigawa State, Nigeria

**Keywords:** Climate change, Perceived impacts, Adaptation, Determinants, Wellbeing, Binary logistic regression, MGA PLS-PM, Pakistan

## Abstract

Rural people, particularly in developing nations, rely on livestock as a key source of income. In Pakistan, rural people depend profoundly on buffalo, cows, sheep, and goats to earn their livelihood. The systems of agricultural production are at risk because of the negative effects of climate change. It badly affects production and quality of milk and meat, animal health, productivity, breeding, feed, and rangelands of livestock production. Climate change risks assessment and adaptation are required to minimize losses from these effects, which are not just technical but also socioeconomically significant. Hence, based on data collected from 1080 livestock herders using a multistage sampling technique in Punjab, Pakistan this study aims to assess perceived impact of climate change on livestock production and to assess coping strategies. In addition, determinants of adaptation strategies and their effects on livestock production was also estimated. Binary Logistic Regression was used to identify the drivers of adaptation strategies. In addition, Multi Group Analysis (MGA) in Partial Least Squares Path Modelling (PLS-PM) was applied to compare adapter and non-adapter of climate change adaptation strategies. Findings indicated that there are spread of various diseases to livestock due to adverse effects of climatic variability. There was reduction in the availability of the livestock's feed. Moreover, competition of water and land resources of livestock was also increasing. Low production efficiency resulted in decline of milk yield and meat production. Likewise, mortality of livestock, increased in still births, reduction in reproductive performance, decline in animal fertility, longevity, and general fitness, decreased birthing rates, rises in age at foremost calving in beef cattle was also prevailing. There were different adaptation policies used by farmers to handle with climate change and these were influenced by several demographic, socioeconomic, and agronomic aspects. Findings indicated that nexus of risk perception, adaptation plans and their determinants are beneficial to reduce the consequences of climatic variability and it improve the wellbeing of the herders. Risk management system may be created to protect livestock against losses caused by extreme weather events by providing awareness regarding influence of climate change on livestock. Easy and cheaper credit should be provided to the farmers to manage with the vulnerabilities of climate change.

## 1 Introduction

Climate change (CC) and its unpredictability pose a greater threat to developing countries than to rich nations [1,2]. Climate change variability is probable to rise the frequency and severity of life-threatening weather events and natural disasters, such as droughts, floods, rising sea levels, and storms [[3], [4], [5]]. Study by Ahmad and Afzal [6] projected that by 2050, the number of human deaths due to natural disasters could be double in Pakistan. Human and economic losses are frequently caused by floods, which are also a major source of social and political instability [6]. The productivity of agriculture may be negatively affected by even small increases in temperature [[7], [8], [9]]. As a result of disintegrating infrastructure and a growing population, climate change has had a substantial impact on emerging countries like Pakistan [10,11]. The Global Climate Risk Index of 2021, ranked Pakistan as the eighth most climate susceptible country in the world, and the projected temperature rise of 2–3 b y 2050 will have a significant impact on rainfall in Pakistan [12,13].

Extreme drought and food shortages have overwhelmed Pakistan for the last two decades because of the climatic variability. This has led to declining farming livelihoods and worsening rural living conditions [12,14]. Agriculture is the principal source of income for more than 60% of Pakistanis. Agricultural resilience to climate change is critical to rural people's well-being and the country's economic success [6]. The production of livestock can have an enormous influence on alleviating poverty [15,16]. Keeping livestock may bring in money, and for many people living in rural and marginal areas, it's their only source of income. To improve farm resilience and adaptability and maintain rural livelihoods, adaptation is essential since the agricultural output will continue to be the primary source of income for over half of South Asia's population [[17], [18], [19], [20]].

Moreover, eight million Pakistani households have livestock, which provide about a third of their total income [21]. In addition, a third of the country's workforce is employed in the agricultural industry, providing food and raw materials for industrial sector [22]. Livestock production might be boosted by using cutting-edge technology to manage climate-related disasters and qualms [23]. Food availability can be improved, poverty can be reduced, rural livelihoods can be supported, and environmental sustainability can be maintained by implementing these measures [[24], [25], [26]]. Despite this, Pakistan's use of environmentally friendly farming practices and technology is still far below the proposed level [25,26]. Due to ineffective climate policy and a lack of financial capacity, the current support system for CC adaptation and mitigation is dreadful [27]. As a result, the livestock sector must have an integrated policy to cope with CC variability.

In Pakistan, 60.54% of agricultural and 11.22% of the country's overall GDP are ascribed to livestock [28]. Pakistan is the world's fourth-largest milk producer, after China, India, and the United States, with a shortfall of 4.57 billion liters a year due to domestic demand. Mostly of livestock herders are landless, limiting their access to traditional rearing of animals [[29], [30], [31]]. Nearly 30 million people are employed in the livestock industry of Pakistan [32]. In Pakistan, only 75% of cattle have access to essential nutrients. In addition, 60% of livestock are deficient in digestible crude protein, and roughly 50% of livestock productivity may be enhanced by increasing the amount of forage [33]. Concerns about environmental changes and their effect on livestock are high in Pakistan since livestock is its primary agricultural industry [22]. Different studies [[34], [35], [36]] outlined that CC, prevalence of new diseases, heat strokes, and livestock losses are all interconnected.

Floods and drought had devastating natural conditions in Pakistan over the past two decades, affecting livestock output and herders livelihoods [12]. However, pastoralists rely on important natural resources such as migration sites, grazing area, and pasture for their livelihoods and livestock rearing is an important source of revenue and sustenance for pastoralists [37,38].

Grazing pastures have been ruined by human intrusion and encroachment, as the population has expanded [39,40]. More severely affected by drought are pastoralists' livelihoods when pasture and water are in short supply [41,42], which reduces livestock beef and milk production, directly and indirectly, leading to the problem of food insecurity [42]. Because of these factors, livestock herders are particularly vulnerable to economic recessions. Long-term drought worsens pastoral livelihoods by increasing the number of livestock deaths and decreasing the quality of animals [43].

It's possible to find an inclusive views in the scientific literature about the influence of climate change on livestock and herders' livelihoods. A plethora of research has looked at, for example, how climate change impacts livestock production, pastoralists' perceptions and adaptation mechanism [44,45]. Despite several studies on the impact of climate change on livestock, little study has been done in Pakistan. Herders' opinions about climate hazards and how they might mitigate the effects of climate change are required to be studied to improve wellbeing of the herders. Climate change is expected to negatively influence livestock productivity in Punjab, Pakistan. Therefore, this study's goal is to determine how farmers observe the impact of climate change on livestock and to examine its consequences in Punjab, Pakistan. In addition, to inspect the farmers' adaptation measures in the study region to mitigate adverse effects of CC. Furthermore, the study also identifies factors that influence adaption drivers and assessing their impact on livestock production. The following sections outline the structure of this research paper. Section 2 explains the research technique. Section 3 elaborate the study's empirical findings and discussion, while section 4 presents a conclusion, as well as some policy implications, of those findings.

## 2 Materials and methods

### 2.1 Data

**Fig. 1:**
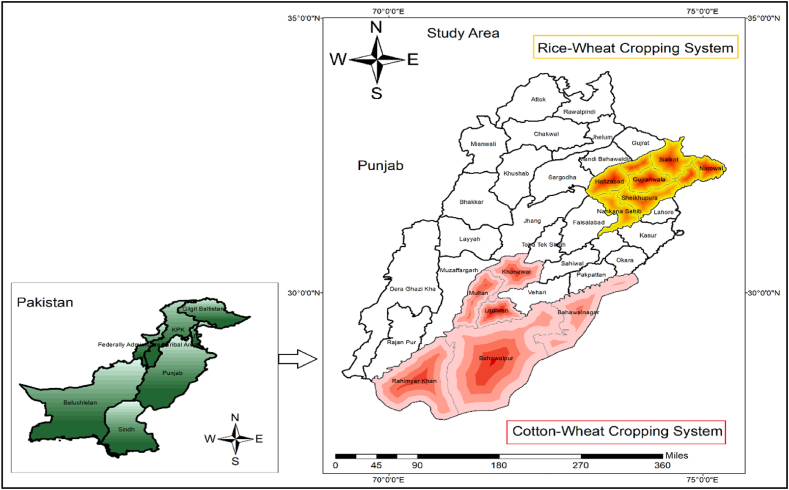
Map of the study area.

As a result of a series of floods (2010–2014), a prolonged period of drought, and severe heat waves, Punjab province has lost significant amounts of its critical crops and livestock during the previous twenty years [46]. Punjab province is Pakistan's most important agricultural area, accounting for 56% of the total arable land and 33% of GDP [47]. Pakistan's agriculture generates 22.7% of GDP and employs 37.4% of the workforce. Around 61.9% of agriculture's value-added and 14. Percent of the nation's GDP is provided by livestock. Nearly a third of the nation's agricultural households are involved in livestock farming, which accounts for more than 8 million families [48,49]. The study was carried out in Pakistan's Punjab province. Based on weather and agroecological cropping patterns, two critical cropping systems (cotton wheat and rice wheat) were selected for study purpose (see Fig. 1). Data on socioeconomic, perceived risks of climate change, adaptation and determinants, and the consequences of climate change adaptation were collected in a well-structured questionnaire. A pre-survey was also conducted to ensure all the necessary information was included.

**Fig. 2:**
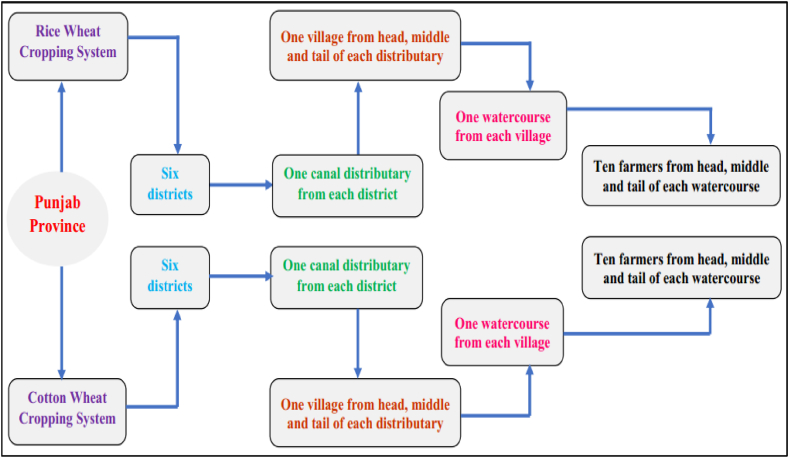
Sampling framework of the study for the selection of the livestock farmers.

During September–October 2020 and February–March 2021, a field survey was undertaken to gather information from livestock herders. Sample herders in the research region were selected using a multistage sampling method (see Fig. 2). Cow, buffalo, goat, and sheep are the most common livestock in Pakistan [12,50], so, the effect of climate change on these animals was studies. Punjab province was designated due to its livestock's’ importance and fast changes in patterns of climate in stage one. Secondly, the cotton wheat cropping system and rice wheat cropping systems were selected. In stage three, six districts were targeted from each system. Stage four was the selection of major canal distributary irrigated to the selected districts. From each distributary one village from the head, middle, and tail was carefully chosen during the fifth stage. During the sixth stage, one watercourse from each village was selected. The final stage was the selection of 10 livestock farmers from the head, middle and tail of the watercourse. Overall, 1080 farmers were interviewed for the study purpose. The sample in the two-time intervals was same. The in-person interviews were directed with the assistance of well-trained enumerators who could communicate in the native language of the area being examined.

### 2.2 process of climate change, perceived risks adaptation and wellbeing of livestock herders

**Fig. 3:**
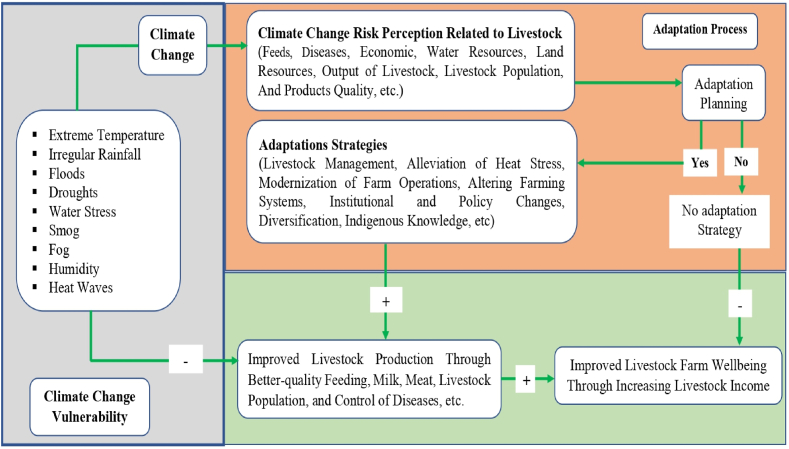
The process of climate change, perceived risks, adaptation and welfare of the livestock farmers.

It's easy to see in Fig. 3 how livestock herders are afraid about the effects of climate change and what they are doing to prepare for them. Farmers' adaptive behaviours need awareness, information, and understanding. Agricultural risk management and effective adaptation technologies are primary motivators [51]. It is difficult for farmers to acclimatize to climate change, and this decision is impacted by various circumstances [52]. If they are external forces, they might also be internal [[53], [54], [55]]. More study is being done that looks at both the inside and the outside behaviour of a person [[56], [57], [58], [59]] than examinations that look at the inside [[60], [61], [62]]. Farmers' internal features, such as their awareness of and familiarity about climate change, attitudes, observations, worries, and risk perceptions, are crucial requirements for effective adaptation [[63], [64], [65]]. These fundamental stimuli substantially influence farmers' adaptive reactions to managing agricultural hazards [63,[66], [67], [68]]. Farmers' concerns, risk perceptions, and adaptive behaviour are linked, although the outcomes differ. Evidence suggest that danger and anxiety play a substantial role in how well people adjust to changing environments. A 5-point Likert scale was employed for closed questions, and the responses were reported as (1) strongly disagree, (2) disagree, (3) no idea, (4) agree, and (5) strongly agree, respectively.

As climate change badly affects the dairy industry, herders must adapt their practices to the new conditions. By adapting, well-being of herders might be improved [51]. Observing how farmers experience climate change is critical since the climate has changed and will endure to change over time. Future adaptation efforts will benefit significantly [69]. It takes two steps for farmers to acclimatize with climate variabilities: the first is to recognize the shift in the weather patterns, and the next step is to take action [70]. In terms of adaptation it is necessary to alter production and management methods, modify breeding techniques, alter institutional or regulatory frameworks, enhance scientific knowledge, and alter farmer perceptions and their ability to adaption [71]. Adaptation methods must be assessed and tailored according to the geography and livestock system [72]. Perceptions of farmers regarding climate change variability are inclined by their agro-ecological background [69].

### 2.3 Determinants of adaptation strategies adopted by livestock herders

The livestock herders' climate change adaptation is influenced by socioeconomic, demographic, and institutional features [73]. A binary response model was used to investigate the drivers effecting a herders' adaption strategy. Using this model, researchers may study the farmer's decisions and their associated probability. It is assumed that each adaptable strategy is distinct from the others to remove the influence of picking one adaptation method over the other. Adapting to climate change requires herders' first to recognize that the climate is fluctuating and believe that this variation constitutes a threat to their well-being that justifies some active or responsive reaction [74]. A farm family will embrace climate change adaption measures if the perceived advantages of doing so are more significant [75,76]. These advantages may include a rise in agricultural output's, net revenue and a reduction in production risk due to climate change [77]. That is, projected assistances from adaptation are equal to A*, where:

(1) $$A_{i^{*}}=X_{i}\alpha +\varepsilon_{i}$$

In above equation, A × is the latent or unobserved variable; which shows that livestock herder i will elect to adjust to climate change (A_i_ = 1) if probable profits are more than zero (A∗ > 0), and zero otherwise. ϵ is the error term and X represents the set of factors that influence the projected profits of adaptation. So, binary logistic regression model was employed to examine the factors influencing the adoption of climate change adaptation measures.

(2) $$\ln\left[\frac{p}{1-p}\right]=\beta_{0}+\beta_{1}X_{1}+\beta_{2}X_{2}+\beta_{3}X_{3}+....+\beta_{j}X_{j}$$

The likelihood of adopting an adaptation strategy is “p” while the likelihood of not adopting an adaptation strategy was 1-p. The regression coefficients of adaptation determinants were β_1_, β_2_ … β_j_. In addition, X_1_, X_2_, …X_j_ represented set of explanatory variables. The intercept was β_0_.

### 2.4 Benefits of climate change adaptation strategies used by livestock herders

It's impossible to get all the answers by focusing on factors that influence climate change adaptation alone. Because of this, it is essential to examine the advantages of farmers' climate change adaptation plans [78]. Smallholder farmers employ multiple strategies to gain additional advantages, including greater agricultural output, climate resilience, and improved livelihoods [[79], [80], [81], [82]]. It is essential to understand how farmers' field approaches in response to long-term environmental changes impact crop and livestock productivity. The productivity of farm households that have used these adaptation measures should be examined to see if they have benefited from the increased output. When it comes to developing methods for adaptability, this is critical. Different studies [[83], [84], [85], [86], [87], [88]] employed propensity score matching (PSM) to evaluate the adaption impacts on the farmers' well-being. Many studies have employed the two-step Heckman technique, which also assumes a normal distribution for unobserved variables [89]. Other research has also used endogenous switching regression [45,76,[90], [91], [92], [93]]. However, this research used new approach i.e., Multi Group Analysis (MGA) of parameters between groups using Partial Least Squares Path Modelling (PLS-PM) [94]. The MGA of PLS-PM uses non-parametric test to examine the differences between the path coefficients of several groups. Moderation across various connections in a research model may be evaluated using Multi Group Analysis (MGA) employing PLS-PM [95]. It is possible to test preset or priori data groups to evaluate whether there are significant variations among group-specific parameter estimations, e.g., path coefficients [96]. MGA may also be used to look for variation between two identical models that belong to distinct groups, as long as the groups' identities are known in advance. Based on the PLS-PM approach, one may determine whether or not multigroup differences are present [95]. Researchers' capacity to detect essential changes in numerous associations across group-specific outcomes significantly improves when MGA is assessed in PLSPM [97,98]. As a result, this research employed Multi-Group Analysis utilizing Partial Least Squares Path Modelling (PLS-PM) to compare the climate change adaptation methods of adapters and non-adapters.

## 3 Results and discussions

### 3.1 Livestock farmers’ perceived impacts related to climate change

The perceived effects of climatic and natural hazards on livestock indicated a decline in forages productivity, deterioration in feed quality, and changed grazing system and historical pattern of grassland production in CWCS and RWCS. The effects of climate change on feed was significantly higher in CWCS than in RWCS (Table 1). The herders’ perceptions about impacts of climatic variability on livestock diseases indicated a higher incidence of mastitis in livestock, alteration in abundance and activity of disease vectors, and increased perseverance and survival of parasites and pathogens. The T-value and *P*-value showed significantly higher impacts on CWCS than RWCS.

**Table 1:** Impact of climatic variability on livestock.

Item	Cropping System	Mean	S.D.	S.E Mean	T-value	*P*-value
**Feeds**						
Decline in forages productivity	RWCS	3.11	1.032	0.044	−33.819	0.000**
	CWCS	4.79	0.523	0.023		
Worsening of feeds quality, e.g., forage digestibility and herbage	RWCS	3.12	1.014	0.044	−35.175	0.000**
	CWCS	4.81	0.459	0.020		
Changed grazing system and historical pattern of prairie production	RWCS	3.22	2.462	0.106	−15.087	0.000**
	CWCS	4.84	0.394	0.017		
**Diseases**						
Higher occurrence of mastitis in livestock animals	RWCS	3.12	1.031	0.044	−34.088	0.000**
	CWCS	4.79	0.484	0.021		
Variation in profusion and action of ailment vectors e.g., insects	RWCS	3.11	1.015	0.044	−35.162	0.000**
	CWCS	4.81	0.473	0.020		
Rise persistence and survival of parasites and pathogens	RWCS	3.15	1.034	0.044	−34.960	0.000**
	CWCS	4.82	0.401	0.017		
**Economic**						
Increased risk household livelihood	RWCS	3.12	1.032	0.044	−36.308	0.000**
	CWCS	4.84	0.387	0.017		
Reduce cash flow and decrease savings of herders	RWCS	3.12	1.016	0.044	−36.006	0.000**
	CWCS	4.83	0.420	0.018		
Loss of farm profits, e.g., milk and production	RWCS	3.10	1.003	0.043	−37.379	0.000**
	CWCS	4.83	0.388	0.017		
Rise in unemployment and cost of labor in rural areas	RWCS	3.11	1.024	0.044	−35.806	0.000**
	CWCS	4.82	0.424	0.018		

Source: Author's Own Survey Results (2021).

Moreover, different economic impacts were perceived by the sampled herders in RWCS and CWCS. The increased risks of household livelihoods reported were significantly higher in CWCS than RWCS. The mean of climate change effects on reduced cash flow and decreased savings of herders due to climate variability was 3.12 ± 1.016 and 4.83 ± 0.420. In addition, the mean value was 3.10 ± 1.003 and 0.83 ± 0.388 of perceived impacts of the loss of farm profits, e.g., milk production due to variability of climate. Furthermore, there were increases in labor cost and unemployment within rural regions with mean value 3.11 ± 1.04 and 4.82 ± 0.424 in RWCS and CWCS, respectively. Considering the results of t-statistics, it was found that the influence of climate change on feed, diseases, and economic status was more in the cotton-wheat than the rice-wheat areas. It means the cotton-wheat zone was more affected area due to climate change.

The mean value of increased rivalry and struggles to grass and water access was 3.16 ± 1.011 and 4.83 ± 0.381 in RWCS and CWCS, respectively. Moreover, RWCS and CWCS had a mean value of 3.11 ± 0.1.008 and 4.82 ± 0.388 of altered land use allocation for livestock. In addition, declining quantity, and quality of drinking water for livestock mean values were 3.14 ± 0.1.018 and 4.83 ± 0.02 for RWCS and CWCS, respectively. T-statistics depicts that the cotton-wheat cropping zone raised rivalry and struggles to water access and fodder. There was altered land use allocation for livestock and observed deteriorating quality and quantity of drinking water compared to rice-wheat zone (Table 2).

**Table 2:** Impacts of climatic variability on water and land resources of livestock.

Water and Land Resources	Cropping System	Mean	S.D.	S.E Mean	T-value	*P*-value
Rises rivalry and struggles to water access and grass	RWCS	3.16	1.011	0.044	−35.883	0.000**
	CWCS	4.83	0.381	0.016		
Altered land use allocation for livestock	RWCS	3.11	1.008	0.043	−37.015	0.000**
	CWCS	4.83	0.388	0.017		
Declining quantity and quality of drinking water for livestock	RWCS	3.14	1.018	0.044	−35.622	0.000**
	CWCS	4.82	0.402	0.017		

Source: Author's Own Survey Results (2021).

T-value (36.997) showed a negative and significant relationship between decreased milk yield and meat production. The mean value exhibited that farmers of cotton-wheat areas observed more decline in milk yield and meat production (4.83 ±0 .37) compared to the rice-wheat cropping zone (3.12 ± 1.01). While in rice-wheat areas, farmers had low production efficiency (4.65 ±0 .90) compared to the cotton-wheat zone (4.11 ± 1.03). T-statistics (9.002) also confirmed that this difference is significant (Table 3).

**Table 3:** Impact of climatic variability on production of livestock.

Production of Livestock	Cropping System	Mean	S.D.	S.E Mean	T-value	*P*-value
Decline in milk yield and meat production	RWCS	3.12	1.01	0.043	−36.997	0.000**
	CWCS	4.83	0.37	0.016		
Low production efficiency	RWCS	4.65	0.90	0.039	9.002	0.000**
	CWCS	4.11	1.03	0.047		

Source: Author's Own Survey Results (2021).

It was found that livestock mortality was higher in the rice-wheat (4.83 ±0 .40) cropping zone than in the cotton-wheat (3.12 ±0 .98) cropping zone. T-value (37.290) showed a significant difference (Table 4). Similarly, the increase in stillbirths was more in the rice-wheat (4.38 ± 1.17) cropping zone than the cotton-wheat (3.63 ±0 .83) cropping zone. T-value (12.185) showed a significant difference. However, a decline in reproductive performance, e.g., lower conception rate, was more in the rice-wheat cropping zone (3.76 ± 1.09) compared to the cotton-wheat cropping zone (3.59 ± 1.79). T-value (1.90) shows a significant difference between two cropping systems. Likewise, low birthing rates and rises in the age at first calving in beef cattle were more in the cotton-wheat cropping zone (3.34 ± 1.22) than the rice-wheat cropping zone (3.02 ± 1.79). T-value (3.404) also shows a significant difference. The t-value (1.529) shows a non-significant difference in decline in animal fertility, general fitness, and longevity in both cropping zones.

**Table 4:** Impact of climatic variability on livestock population.

Livestock Numbers	Cropping System	Mean	S.D.	S.E Mean	T-value	*P*-value
Mortality of livestock	RWCS	4.83	0.40	0.017	37.290	0.000**
	CWCS	3.12	0.98	0.043		
Increased in still births	RWCS	4.38	1.17	0.050	12.185	0.000**
	CWCS	3.63	0.83	0.036		
Reduction in reproductive performance, e.g., inferior conception rates	RWCS	3.59	1.79	0.077	−1.900	0.058*
	CWCS	3.76	1.09	0.047		
Decline in animal fertility, longevity, and general fitness	RWCS	3.58	1.60	0.069	−1.529	0.127^NS^
	CWCS	3.70	1.04	0.045		
Decreased birth rates, rises in age at first calving in beef cattle	RWCS	3.02	1.79	0.077	−3.404	0.001**
	CWCS	3.34	1.22	0.053		

Source: Author's Own Survey Results (2021).

The results of the farmers' perceived impacts of climatic and natural hazards on livestock product quality indicated a non-significant difference in reduction of milk quality in both cropping zones (Table 5). But, reduction in meat quality was more in the rice-wheat cropping zone (4.31 ± 1.36) than cotton-wheat cropping zone (3.74 ±0 .89).

**Table 5:** Impact of climatic variability on livestock product quality.

Product quality	Cropping System	Mean	S.D.	S.E, Mean	T-value	*P*-value
Observed greater reductions in milk quality	RWCS	3.63	1.77	0.076	−1.447	0.148^NS^
	CWCS	3.75	0.84	0.036		
Observed greater reductions in meat quality	RWCS	4.31	1.36	0.059	8.164	0.000**
	CWCS	3.74	0.89	0.038		

Source: Author's Own Survey Results (2021).

### 3.2 Adaptation strategies to moderate the impact of climate change

Management of livestock farms is essential to get higher income. It has key role in mitigating the adverse effects of CNHs. The 38.1% of the farmers’ in CWCS and 37.67% of RWCS used livestock management strategy to reduce the catastrophes of CNHs. The population of livestock is higher in CWCS than in RWCS. In addition, around 28.7% and 26.9% of sampled respondents in RWCS and CWCS used alleviation of heat stress to reduce the impacts of CNHs. Findings indicate that herders who adopt modernization of farm operations to manage with the hostile effects of climatic and natural hazards. It was estimated that 22.90% of farmers in CWCS and 24.4% in RWCS used the modernization strategy of farm operations. In addition, altering farming systems components was adopted by 29.3% and 28.7% of respondents in RWCS and CWCS, respectively.

Similarly, 21.2% and 24.3% RWCS and CWCS famers adopted institutional and policy changes in response to CNHs adversaries. The farmer uses the diversification of livestock farms and income sources as a sustainable solution to manage the losses caused by CNHs. It was estimated that 27.2% and 29.3% of farmers in RWCS and CWCS used the diversification strategy. It was examined that all indicators of adaptation policies of livestock were different in cotton-wheat cropping zone and rice-wheat such as livestock management, alleviation of heat stress, modernization of farm operations, altering farming systems components, institutional and policy changes, and diversification (Table 6). Indigenous knowledge and spatial adaptation were adopted by 31.82% and 34.39% in RWCS while of 28.43% and 35.52% in CWCS, respectively.

**Table 6:** Adaptation practices of livestock.

Adaptation Practices Livestock	RWCS		CWCS	
	Yes (%)	No (%)	Yes (%)	No (%)
Livestock management	38.1	61.9	37.67	62.33
Alleviation of heat stress	28.7	71.3	26.9	73.1
Modernization of farm operations	24.4	75.6	22.90	77.1
Altering farming systems components	29.3	70.7	28.7	71.3
Institutional and policy changes	21.2	78.8	24.3	75.7
Diversification	27.2	72.8	29.3	70.7
Indigenous knowledge	31.82	68.18	28.43	71.57
Spatial adaptation	34.39	65.61	35.52	64.8

Source: Author's Own Survey Results (2021).

### 3.3 Drivers of climate change adaptation strategies (DCAS)

Effective adaptation measures may be able to reduce livestock losses (Faisal et al., 2020). Climate change has been declining rural livelihoods in Pakistan, causing livestock illnesses, floods, and droughts to become more severe. Many pastoralist families lost animals due to these weather-related catastrophes [6]. Model significance and predictive power for all the hypothesis were tested satisfactorily (Table 7).

**Table 7:** Testing of hypothesis for model predictive power and significance.

Models	Chi-squared	D.F.	*P*-level	Model correctness (%)	−2 Log likelihood	Cox & Snell R Square	Nagelkerke R Square
Livestock management	656.32	16	0.000	82.1	840.840	0.455	0.607
Alleviation of heat stress	299.18	16	0.000	72.5	1188.369	0.242	0.324
Modernization of farm operations	310.97	16	0.000	73.5	1186.192	0.250	0.334
Altering farming systems components	209.43	16	0.000	70.2	1282.413	0.176	0.235
Institutional and policy changes	219.95	16	0.000	69.4	1275.908	0.184	0.246
Diversification	255.83	16	0.000	72.2	1221.574	0.211	0.283
Indigenous Knowledge	221.47	16	0.000	71.2	1271.691	0.185	0.248
Spatial adaptation	679.96	16	0.000	81.9	816.605	0.467	0.623

Source: Author's Own Survey Results (2021).

Age of the farmers had substantial and positive association with adaptation measures such as alleviation of heat stress, modernization of farm operations, altering farming systems components, institutional and policy changes, indigenous knowledge. However, some adaptation strategies associated to crop such as livestock management, diversification, and spatial adaptation had non-significant relation (see Table 8). Younger livestock herders are more likely to involve in CC mitigation techniques than elder ones [25].

**Table 8:** Binary logistic regression: Parameter estimates of climate change adaptation strategies.

Independent Variables	Adaptation Strategies (Dependent variables)															
	Livestock management		Alleviation of heat stress		Modernization of farm operations		Altering farming systems components		Institutional and policy changes		Diversification		Indigenous Knowledge		Spatial adaptation	
	B	Sig.	B	Sig.	B	Sig.	B	Sig.	B	Sig.	B	Sig.	B	Sig.	B	Sig.
Age (years)	.014	.085	.015	.018	.017	.011	.018	.004	.021	.001	.007	.246	.017	.005	.014	.084
Education (years)	.233	.000	.094	.000	.113	.000	.099	.000	.098	.000	.084	.000	.098	.000	.216	.000
Crops and Dairy farming experience (years)	.045	.000	.025	.000	.020	.002	.023	.000	.023	.000	.025	.000	.022	.000	.042	.000
Veterinary hospital distance (Kilometer)	.013	.304	.001	.955	.005	.656	.009	.353	.013	.198	.013	.215	.010	.306	.014	.279
Animal inventory/Animal number	.162	.000	.025	.119	−.012	.438	.067	.000	.016	.280	.024	.124	.065	.000	.166	.000
information on weather forecasting	.616	.002	−.248	.099	.466	.004	−.194	.213	−.129	.402	.341	.045	−.153	.329	.790	.000
Information on climatic and natural hazards	.019	.951	−1.688	.000	−.132	.594	−.181	.455	.167	.542	−.308	.275	−.175	.471	.175	.567
Livestock-related training/workshop Dummy	1.013	.000	.212	.179	.227	.131	.379	.010	.185	.213	.164	.283	.391	.008	1.170	.000
Household size	.249	.000	.112	.003	.119	.002	.095	.009	.083	.024	.076	.038	.105	.004	.222	.000
Market access information	−.137	.641	.440	.064	.049	.835	−.254	.274	−.106	.593	1.563	.000	−.211	.365	.076	.797
Credit access	−.133	.656	1.671	.000	.477	.048	.153	.516	.696	.010	.635	.021	.143	.545	−.135	.653
Livestock labor force No.	.326	.000	.137	.000	.135	.000	.118	.000	.114	.000	.113	.000	.142	.000	.395	.000
Agricultural extension services provided for livestock production	.391	.038	.266	.090	.223	.150	−.009	.951	.023	.879	−.172	.267	.018	.908	.273	.153
Dummy Cropping System (D = 1 if Rice-Wheat Cropping Zone; 0 = otherwise)	.076	.716	.159	.370	−.241	.160	.044	.795	.490	.007	.086	.633	.030	.857	−.072	.734
Dummy Remittances	.379	.224	.678	.009	.421	.096	.197	.430	.139	.565	−1.045	.000	.197	.432	.538	.087
Dummy Off Farm Income	.788	.007	.724	.004	.397	.093	.570	.015	.359	.138	.312	.164	.552	.019	.439	.134
Constant	−9.925	.000	−4.832	.000	−4.241	.000	−4.595	.000	−5.223	.000	−4.091	.000	−4.759	.000	−9.835	.000

Source: Author's Own Survey Results (2021).

There was a positive and significant relationship with years of schooling of the herders and adaptation strategies. It means, educated farmers had more adaptations strategies related to livestock as compared to uneducated farmers. Education is a significant component in developing the adoption phenomena [99]. The number of years a person has spent in school improves the likelihood of adjusting to CC [25]. Crops and dairy farming experience of the herders had also positive significant association with all adaptation strategies. It means, experienced herders had more adaptations strategies related to livestock as compared to those farmers who had little farming experience. The outcome of this variable is reliable with the findings of Shahbaz et al. [100], livestock experience is a key important social variables impacting the methods employed by small livestock farms. Additionally, livestock rearing expertise is a critical social component impacting the adaptation techniques employed by small herders. Older livestock herders are more conservative, old-style, and unimaginative than younger livestock farmers, and as a result, they are less receptive to innovative methods. The old age herders are more risk-averse than younger and inexperienced small livestock holding herders.

Distance from veterinary hospital had non-significant association with adaptation measured undertaken by the herders. It means that distance from hospital had no impact on the adaptation strategies related to livestock to abate the effect of CC. The distance between a livestock farm and a veterinary facility is also one of the most important factors influencing livestock farmers' adoption of CC mitigation techniques [100]. Animal inventory had significant and positive relation with some adaptation strategies related to livestock such as livestock management, altering farming systems components, and indigenous knowledge and spatial adaptation. While remaining variables such as alleviation of heat stress, modernization of farm operations, institutional and policy changes and diversification had non-significant relation with animal inventory. Animal ownership is a significant economic indicator that impacts the tactics that are implemented in this industry. So, larger livestock herders have more resources to spend in farming than smaller. They may therefore implement a greater variety of solutions to combat climate change than their smaller livestock counterparts. The overall number of animals is just as significant as the entire agricultural area available for crop production (Shahbaz et al., 2020 [93]).

The knowledge regarding weather anticipation had significant and positive association with adaptation strategies such as livestock management, modernization of farm operations, diversification, and spatial adaptation. However, information on climate predicting had non-significant relation with alleviation of heat stress, altering farming systems components, institutional and policy changes, indigenous knowledge. The daily and seasonal forecasting of weather (i.e., precipitation and temperature) has a favorable and statistically significant impact on the techniques of adapting to CC. According to Abid et al. [12], familiarity on weather projecting improves the likelihood of adaption to CC.

Climatic and natural hazards information had significant and negative relation with alleviation of heat stress. All other adaptation mechanisms connected to livestock, on the other hand, contained non-significant information on their relationship to climate and natural disasters. Climate knowledge is one of the most important aspects that determine the methods that are used on livestock farms. It is crucial aspect in adopting climate strategies because if livestock herders are aware of patterns of climate change, they will be able to take appropriate steps [47]. Climate understanding and the methods that have been implemented are inextricably linked [51,101].

It was found that livestock-related training/workshop had significant and positive relation with livestock management, Altering farming systems components, and indigenous knowledge and spatial adaptation. It means training had positive impact on adaptation of livestock management, altering farming systems components, and indigenous knowledge and spatial adaptation. Herders who have received training are more conscious of CC impact than who have not received instruction through training program and workshops [102,103].

Household size is positively related with all adaptation strategies. It means, if farmers had large family size then their adaptation level were also high. The size of a herder family is an imperative social factor that can influence DCAS. The majority of adaptation measures adopted by herders to combat climate change required large scale labor. Family size of the herders is vital social aspects affecting DCAS [104]. As a result, a large family size can positively impact CC adaptation techniques [105]. Market access information had positive and significant association with alleviation of heat stress and diversification. But, all other adaptation approaches related to livestock had non-significant relation with market access information.

The impact of credit access had positive and significant link with four out of eight adaptation strategies related to livestock such as alleviation of heat stress, modernization of farm operations, institutional and policy changes, and diversification. While remaining four adaptation strategies such as livestock management, altering farming systems components, indigenous knowledge, and spatial adaptation had non-significant relation with credit access. Credit accessibility delivers herders an opportunity to make up for the lack of cash in their businesses [100]. Credit empowers farmers to increase acquisition of their capital to obtain the inputs they require for production. Herders are also encouraged to embrace new technology or improve existing ones to increase production [106]. Furthermore, access to financing allows farmers to raise their output while also becoming more efficient [[107], [108], [109]].

There was positive and significant association of livestock labor force with all adaptation strategies used by the farmers. It means, if the farmers had large livestock labor force then their adaptation level were also high. The livestock labor force is also a crucial factor of adaptation techniques worldwide, but notably in developing nations since most of the strategies implemented are labor-intensive. As a result, increasing the number of livestock laborers in developing nations helps raise the level of adaptation to CC [105].

All indicators of adaptation strategies had non-significant relation with agricultural extension services excepted livestock management. According to the study's findings done by Deressa et al. [110], there is a direct association between the adoption of CC policies and the use of extension services. All indicators of adaptation strategies related to livestock had non-significant relation with cropping system excepted institutional and policy changes. Remittances had significant and positive relation with alleviation of heat stress, while significant and negative relation with diversification. But, all other indicators of adaptation approaches had non-significant association with remittances. Income from out-migration, small rural enterprises, tourism, wages of labor, and the harvesting of medicinal herbs and plants all grant to the livelihood and food security of the rural poor in emerging countries [22]. Poudel et al. [111], indicated that remittances from migrants have the potential to benefit local small firms that employ both skilled and unskilled workers.

Off farm income access had significant and positive link with four out of eight adaptation strategies such as livestock management, alleviation of heat stress, altering farming systems components and indigenous knowledge. While remaining four adaptation strategies related to livestock such as modernization of farm operations, institutional and policy changes, diversification and spatial adaptation relation with off farm income. Following the findings of Abbas et al. [23], the livestock herders' off-farm earning activities had a favorable influence on the climate adaption measures.

### 3.4 Effect of climate change adaptation measures on livestock herders’ wellbeing: rice wheat cropping system

The results of SmartPLS showed that in the Rice Wheat Cropping System (RWCS) use of livestock management to moderate the outcome of climate change. The coefficient of adapter and non-adapter was −0.168 and −0.386, while overall, attained a reduction of −21.80% due to the use of livestock management. The livestock herder who adopted alleviation of heat stress to reduce the adversaries of CC has −19.20% less impact than the non-adapter. Moreover, the adapter of modernization of farm operations and altering farming systems components to cope with CC has −21.20% and −23.20% less impact as compared to their counterpart who was non-adapter. Similarly, the study's result designated a reduction of −9.40% and −20.80% of adapters that use the strategies of institutional and policy changes and livestock diversification than that of non-adapter (see Table 9).

**Table 9:** Effect of climate change adaptation measures on RWCS.

Adaptation Measure	Path Coefficients (non-adapter)	Path Coefficients (adapter)	**p-Value (non-adapter)**	p-Value (adapter)	Impact Reduction %
Livestock management	−0.386	−0.168	0.000	0.000	−21.80%
Alleviation of heat stress	−0.366	−0.174	0.000	0.001	−19.20%
Modernization of farm operations	−0.367	−0.155	0.000	0.002	−21.20%
Altering farming systems components	−0.370	−0.138	0.000	0.047	−23.20%
Institutional and policy changes	−0.358	−0.164	0.000	0.004	−19.40%
Diversification	−0.345	−0.137	0.000	0.0023	−20.80%

Source: Author's Own Survey Results (2021).

### 3.5 Effect of climate change adaptation measures on livestock herders’ wellbeing: cotton wheat cropping system

In the Cotton Wheat Cropping System (CWCS), the coefficients of all the adaptation measures of adapter and non-adapter were significant. Overall, there was a significant reduction in the effects CC of all the adaptation strategies of the adapter. There was −9.10% less impact of CC to the farmers using livestock management than the non-adapter. Meanwhile, −31.90% reductions in the impact of CC were estimated due to the adapter's alleviation of heat stress. The farmers that use modernization of farm operations have a coefficient of −0.270 and non-adapter −0.355 with a difference of −8.50% between the two groups. The difference between the livestock herders that use shifting farming systems components and those who don't adopt was −22.60%. Moreover, a difference in the impacts of CC was estimated at −20.10% due to institutional policy changes and livestock diversification (see Table 10).

**Table 10:** Effect of climate change adaptation measures on CWCS.

Adaptation Measure	Path Coefficients (non-adapter)	Path Coefficients (adapter)	p-Value (non-adapter)	p-Value (adapter)	Impact Reduction %
Livestock management	−0.359	−0.268	0.000	0.000	−9.10%
Alleviation of heat stress	−0.507	−0.188	0.000	0.019	−31.90%
Modernization of farm operations	−0.355	−0.270	0.000	0.000	−8.50%
Altering farming systems components	−0.431	−0.205	0.000	0.003	−22.60%
Institutional and policy changes	−0.416	−0.215	0.000	0.016	−20.10%
Diversification	−0.419	−0.218	0.000	0.005	−20.10%

Source: Author's Own Survey Results (2021).

### 3.6 Discussion

Climate change has less impact on mixed-crop livestock producers who use mitigation practices to abate climatic vulnerabilities. As a result, their crop-livestock interaction, output, and farm revenue are higher, and they have greater market flexibility [112]. For example, according to the findings of Karimi et al. [19,20], the current livestock production methods should be revisited in light of new information on sustainable rangeland management and participatory production knowledge to decrease the negative impact of CC on wellbeing of livestock herders. In addition, prevailing rules of livestock management should be improved to adapt to CC conditions. Because of a lack of climate change adaptation methods, livestock output is dropping significantly [113]. According to Joshi et al. [114], research, farmers and scientists should have access to CC adaptation information for the benefit of livestock farms.

By promoting livestock farming, we can improve our country's food security [115]. Women's engrossment in management of livestock farming and its effects on their livelihood was examined by Manzoor et al., [116]. Female livestock workers clean sheds and make dung pads, which is primarily a woman's job. Women face complications in attending family gatherings, getting to know relatives, and participating in social activities when working with cattle. According to Ali [24], livestock herders should be made aware of climatic risks and coping practices to lower the impact of CC on their livelihood. Involving young people in livestock farming and expanding the size of the farm might enhance household income [117]. Similarly Mahmood et al. [116] revealed that livestock farming could provide the fundamental needs of households. Furthermore, the money made from the livestock industry is used to benefit the farms' health, education, and housing, all of which help to improve the farmers' level of living.

To deal with climatic hazards, people adopt traditional coping measures such as mixed farming, livestock diversification, increased drinking water availability, reduced animal population, and the planting of trees and mud on their roofs and floors. Various factors influenced the adoption of climate change coping techniques, including awareness about climate change, animal inventories, previous livestock farming experience, livestock training and workshops, distance from a veterinarian facility, and livestock working hours [100].

According to research Ali [21,84], livestock herders' adaption tactics are often better handled. By boosting milk and butter output, the amount of land dedicated to feeding can be lowered. Livestock insurance benefits milk output significantly while having unfavorable influence on poverty levels. Herders who sold cattle to help alleviate the influence of climate change saw a reduction in milk output, but household income increased, and poverty declined as a result. Climate change appears to be having a detrimental impact on both output and revenue of the livestock.

## 4 Conclusion and policy recommendations

In developing countries like Pakistan, about 65–70% population lives in rural areas. Agriculture is the major means of livelihood and food security for the rural population. In Pakistan, rural people depend profoundly on buffalo, cows, sheep, and goats as their prime source of livelihood. Agricultural production systems are at risk because of the negative impacts of climate change. It is affecting milk production and quality, animal health and productivity, breeding, feed crops, and rangelands of livestock production. This study determined herders perceived impact of CC on livestock. The herder's adaptation options to offset the effects of CC on livestock were also identified. In addition, impacts of climate change adaption strategies and their drivers were also determined. The findings designated that there are spread of various diseases to livestock due to climate change. There was decline in the availability of the livestock's feed. Moreover, competition of water and land resources of livestock was also increasing. Low production efficiency caused in decline of milk yield and meat production. Likewise, mortality of livestock, increased in still births, reduction in reproductive performance, decline in animal fertility, longevity, and general fitness, decreased birth rates, rises in age at first calving in beef cattle was also prevailing due to climate change. Finally, findings indicated that relationship risk perception, adaptation measures and determinants of climate change are beneficial to moderate the effects of climatic unpredictability and it accelerate the wellbeing of the livestock herders.

Management of livestock farms is essential to get higher income and improve rural livelihoods. It has key role in mitigating the adverse effects of CC. The different adaptation strategies used by herders were livestock management, alleviation of heat stress, modernization of farm operations, altering farming systems components, institutional and policy changes, diversification, indigenous knowledge, and spatial adaptation. The results of SmartPLS indicated that the use of adaptation measures had significant effect in moderation of climate change negative effects. Climate change has less impact on mixed-crop livestock producers who use mitigation techniques to minimize climatic vulnerabilities. As a result, their crop-livestock interaction, output, and farm revenue are higher, and they have greater market flexibility.

The use of social media and extension services should be enhanced across the country, particularly in the RWCS and CWCS, to convey precise information and promote awareness to livestock farmers. Mass media and information technology should encourage herders' understanding of climate change. Further IT industry may assist farm households in raising climate change awareness in the country. Risk management system may be created to protect livestock against failures caused by extreme weather events. Policies that boost feeding of livestock among resource-strapped rural poor people can be accomplished through official and casual training. This is critical for knowledge transmission and cooperation in providing the resources required to implement these methods. As majority of the herders were uninformed of illness onset, transmission, spillover and persistence, which caused in livestock losses, new approaches for detecting, responding to, and controlling infectious diseases should be developed. Develop and introduce higher-productivity cattle breeds that are less vulnerable to climate change's inescapable effects. Different adoption patterns imply that educational programs for livestock herders should be modified to encourage them to use optimum combinations of methods rather than relying on a single strategy. Increasing the nutritional value of cattle feed is the need of the time. Easy and cheaper credit should be ensured to the farmers to cope the negative effects of climatic vulnerabilities.

## Limitation and future guidelines

This research was carried out in Punjab, Pakistan, so that future studies may be directed in other provinces of Pakistan. Moreover, secondary data may also be used to support the results. In addition, the impact of recent floods can also be examined on livestock.

## Author contribution statement

Conceived and designed the experiments; Muhammad Usman and Asghar Ali.

Performed the experiments; Sajjad Ahmad Baig and Rimsha Akram:

Analyzed and interpreted the data; Muhammad Usman and Abdulazeez Hudu Wudil:

Contributed reagents, materials, analysis tools or data; Joanna Rosak-Szyrocka, Ladislav Pilař and Muhammad Usman.

Wrote the paper: Muhammad Usman and Asghar Ali.

## Data availability statement

Data will be made available on request.

## Declaration of competing interest

The authors declare that they have no known competing financial interests or personal relationships that could have appeared to influence the work reported in this paper.
